# The Relationship Between Cytokine Concentrations and Severity Scoring Index for Crimean-Congo Hemorrhagic Fever

**DOI:** 10.7759/cureus.34882

**Published:** 2023-02-11

**Authors:** Sevda Onuk, Hilal Sipahioglu, Zehra Beştepe Dursun, Esma Eren, Hatice Aslan Sırakaya, Sibel Kuzugüden, Ilhami Celik

**Affiliations:** 1 Department of Intensive Care Unit, Kayseri City Education and Research Hospital, Kayseri, TUR; 2 Department of Clinical Microbiology and Infectious Diseases, Kayseri City Education and Research Hospital, Kayseri, TUR; 3 Department of Internal Medicine, Kayseri City Education and Research Hospital, Kayseri, TUR; 4 Department of Clinical Biochemistry, Kayseri City Education and Research Hospital, Kayseri, TUR

**Keywords:** interleukin, hmgb1, il-6, il-10, ssi, cytokine, il-1β, tumour necrosis factor-α (tnf-α), crimean-congo hemorrhagic fever virus

## Abstract

Background

This study aimed to investigate the effects of serum high mobility group box-1 (HMGB1), interleukin (IL)-6, IL-8, IL-1β, IL-10, and tumor necrosis factor alpha (TNF-α) levels on disease severity and mortality in Crimean-Congo hemorrhagic fever (CCHF) patients.

Materials and methods

This study was performed prospectively in the intensive care unit (ICU) and infection ward of a tertiary hospital in the Republic of Türkiye. Patients aged 18 years and older diagnosed with CCHF were included.

Results

Our study included 30 patients, of whom 83.3% were male, where the mean age was 51.6±14.35 years. The most common clinical findings in patients were malaise (90%) and myalgia (63.3%). In our study, IL-1β levels were found to be 1173.6 (783.0-1823.0) pg/mL, IL-6 69.9 (56.8-133.1) pg/mL, IL-8 191.2 (152.8-516.9) pg/mL, TNF-α 129.5 (104.9-270.8), HMGB1 37.01 (29.26-75.18), and IL-10 190.1 (IQR: 147.8-387.8) pg/mL. The patients' median Severity Scoring Index (SSI) score was found to be 2.5 (1.8-5.5). There was a moderate correlation between the patients' SSI score and serum IL-6 (r=0.464, p=0.010), TNF-α (r=0.420, p=0.021), and IL-10 levels (r=0.518, p=0.003), and a weak correlation between serum HMGB1 (r=0.392, p=0.032). The correlation between SSI and creatine phosphokinase (CPK) levels (r=0.499, p=0.036) was observed to be moderate.

Conclusion

It was seen that IL-10, IL-6, TNF-α, HMBG-1, and CPK levels evaluated at the CCHF patients' time of admission to the clinic and SSI clinical score were found to be significantly related. It is clear that more studies with patients and groups of healthy volunteers are needed on this subject.

## Introduction

People can be afflicted by many viral diseases, of these, Crimean-Congo hemorrhagic fever (CCHF) is perhaps the most dangerous. Before the onset of hemorrhagic syndrome, nonspecific signs and symptoms tend to present. A mortality rate of 5-30% has been reported in general, with variations depending on clinical care facilities and patient characteristics [1].

Where cases are critical, hemorrhagic signs occur some three to six days after the emergence of symptoms [2]. Mortality rates are high, with geographic area and standards of healthcare having a marked impact on outcomes. Furthermore, there is an inverse relationship between the number of patients and the mortality rate in some reports where the number of patients is low, with the opposite presenting in larger-scale studies. In Türkiye, 4.8% is the observed average rate of mortality [3].

CCHF may cause endothelial injury. Therefore, microvessel damage and disturbance of hemostasis may develop [1]. Tumor necrosis factor-alpha (TNF-α) is held to be critical with respect to the activation of endothelial cells from CCHF virus-infected dendritic cells [4,5]

Studies have shown that where proinflammatory cytokine serum levels and TNF-α, interleukin (IL)-8, IL-6, etc. are high, there is an association with disease severity in CCHF patients [6,7]. CCHF-infected dendritic cells (DCs) and macrophages are also seen to release these cytokines [4,5].

These findings suggest that coagulation disorders and, indirectly, fatal bleeding in CCHF patients are probably the results of the increase in pro-inflammatory cytokine levels. The goal of this study, therefore, was to investigate how serum TNF-α IL-1β, IL-8, IL-10, IL-6, and serum high mobility group box-1 (HMGB1) levels affect disease severity and mortality in CCHF patients.

## Materials and methods

In our study, 30 patients who were diagnosed with CCHF, according to the CCHF guidelines, were included. This study obtained ethical approval from Kayseri City Education and Research Hospital Ethical Board (Approval number: 443/14.07.2021 dated July 14, 2021). The study was conducted in accordance with the ethical standards laid down in the 1964 Declaration of Helsinki and its later amendments. The study was conducted in Kayseri City Training and Research Hospital, Kayseri, Türkiye.

Patients

In our study, patients aged 18 years and older, who were hospitalized for 1-12 days from the start of symptoms (malaise, myalgia, nausea, headache, diarrhea, bleeding, petechia, etc.) and where the polymerase chain reaction (PCR) test had confirmed infection, were included. Since it will affect the clinical course and cause a change in cytokine levels, patients with an active cancer diagnosis, liver and kidney failure, and who are pregnant, were excluded. In addition, patients using drugs that affect cytokine levels (such as corticosteroids, TNF, and/or interleukin antagonists and interferon) were also excluded from the study. Patient medical cards, demographic data, laboratory results, and clinical outcome characteristics were evaluated.

Identification of suspected CCHF cases

For the purposes of this study, a CCHF case was held to be primary if there was laboratory confirmation of the case within a particular region or community. A CCHF case was held to be secondary if there was a reference laboratory-confirmed CCHF case where close contact had occurred with the primary case within 14 days of disease onset. Diagnosis of CCHF was confirmed by positive reverse transcription-polymerase chain reaction (RT-PCR) test and/or positive IgM antibodies by enzyme-linked immunosorbent assay (ELISA).

Cytokine level determination

Patients with CCHF were asked to provide samples of both plasma and serum on the day of hospitalization. These were then stored at -80° C. Supernatants from sera separated as described were tested for TNF-a, IL-6, IL-8, and IL-1β concentrations (Bioassay Technology Laboratory, Shanghai, China) with the enzyme-linked immunosorbent. The concentration of IL-1β, IL-6, IL-8, TNF-α, HMGB-1 proteins, and IL-10 were determined using commercial sandwich ELISA kits according to the manufacturer's instructions (Bioassay Technology Laboratory; E0143Hu, E0089Hu, E0082Hu, E0099Hu, E1635Hu, and E0119Hu, respectively). ELISA Reader (BioTek Instruments, Inc., Winooski, Vermont, United States) was used to measure cytokine levels using the Kc Junior Software (Bio-Tek Instruments, Inc.).

Severity score index (SSI)

The SSI was calculated from the laboratory and clinical evaluation results of all patients included in our study, which was evaluated during their hospitalization. The components of the SSI score were: platelet count (0-3 points), activated partial thromboplastin time (aPTT) (0-3 points), fibrinogen (0-3 points), bleeding (0-3 points), and somnolence (0 or 1 point). The patients were classified according to their SSIs (mild, moderate, and severe) and evaluated upon first admission [8].

Statistical analysis

IBM SPSS Statistics for Windows, Version 25.0 (Released 2017; IBM Corp., Armonk, New York, United States) was used for the requisite statistical analysis. The Shapiro-Wilk test was used to determine univariate data conformity to the normal distribution, multivariate normal distribution conformity was ascertained via the Dornik and Hansen omnibus test, and the homogeneity of variance with the Levene test. According to the data's normal distribution and variance homogeneity, appropriate parametric and nonparametric analyses were used. Quantitative variables are presented as mean±SD and median range (maximum-minimum) with n (%) representing categorical variables. A 95% CI was used in respect of the variables to be analyzed, and a p-value not exceeding 0.05 was held to be significant.

## Results

The study included 30 patients, of which 83.3% were male, and the mean age was 51.6±14.35 years. The patients’ demographic and clinical data are set out in Table 1.

**Table 1:** Demographic and clinical findings of CCHF patients SSI: severity score index; IQR: interquartile range; CAD: coronary artery disease; MV: mechanical ventilation; CCHF: Crimean-Congo hemorrhagic fever

Demographic findings	Total (n=30)
Age (years), mean ± SD	51.6±14.35
Sex (male), n (%)	25 (83.3)
BMI (kg/m^2^), mean ± SD	26.1±2.88
Time elapsed from the tick bite to the onset of symptoms (days), mean ± SD	4.3±2.88
Time elapsed from symptom onset to hospitalization (days), median (IQR)	1.5 (1.0-4.0)
Time elapsed from the tick bite to hospitalization (days), mean ± SD	5.3±2.81
SSI score, median (IQR)	2.5 (1.8-5.5)
Comorbidity, n (%)	
Hypertension	2 (6.7)
Liver disease	1 (3.3)
Hematological disease	1 (3.3)
Diabetes	1 (3.3)
CAD	1 (3.3)
Classification of SSI score, n (%)	
Mild	15 (50.0)
Moderate	12 (40.0)
Severe	3 (10.0)
Clinical outcome	
Bleeding, n (%)	10 (33.3)
Need for MV, n (%)	3 (10.0)
Duration of ICU hospitalization (days), median (IQR)	0.5 (0-8.0)
Duration of hospital stay (dys), median (IQR)	7.0 (4.0-10.0)
Mortality, n (%)	4 (13.3)

With respect to clinical findings, the most common reported were malaise (90%) and myalgia (63.3%). Bleeding was present in 10 (33.3%) patients and seven (23.3%) had bleeding in more than one site (Table 2).

**Table 2:** Symptoms of CCHF patients CCHF: Crimean-Congo hemorrhagic fever

Symptoms	n (%)
Malaise	27 (90.0)
Myalgia	19 (63.3)
Nausea	11 (36.7)
Headache	10 (33.3)
Diarrhea	10 (33.3)
Bleeding in more than one area	7 (23.3)
Petechia	6 (20.0)
Vomiting	5 (16.7)
Abdominal pain	4 (13.3)
Somnolence	4 (13.3)
Impaired consciousness	3 (10.0)
Respiratory symptoms	2 (6.7)
Bleeding gums	2 (6.7)
Ecchymosis	2 (6.7)
Hematuria	2 (6.7)
Hematoma	2 (6.7)
Melena	1 (3.3)
Hematemesis	1 (3.3)
Hemoptysis	1 (3.3)
Epitaxis	1 (3.3)
Hepatomegaly	1 (3.3)
Splenomegaly	1 (3.3)

The patients' mean platelet count was found to be 90.1±65.08 /mm^3^. The patients' median aspartate transaminase (AST), alanine transaminase (ALT), and lactate dehydrogenase (LDH) values were reported as 148.5 (43.3-378.0), 101.0 (33.5-256.0) and 443.0 (305.0-704.5) IU/L, respectively. The patients' median CPK value was found to be 222.0 (79.3-339.3). The patients' detailed biochemical values are shown in Table 3.

**Table 3:** Laboratory results WBC: white blood cells; IQR: interquartile range; MPV: mean platelet volume; AST: aspartate aminotransferase; ALT: alanine aminıtransferase; fL: femtoliter; IU: international unit; L: liter; LDH: lactate dehydrogenase; CPK: creatine phosphokinase; GGT: g-glutamyl transferase; BUN: blood urea nitrogen; CRP: C-reactive protein; PT: prothrombine time; ALP: alkaline phosphatase; INR: international normalized ratio; aPTT: activated partial thromboplastin clotting time

	Total (n=30)	Reference range
WBC (10^3^/µL), median (IQR)	3.27 (2.06-5.64)	4,5-10
Neutrophil (%), mean ± SD	58.5±15.53	41-75
Lymphocyte (%), mean ± SD	31.4±14.08	12-48
Platelet (10^3^/µL), mean ± SD	90.1±65.08	150-450
MPV (fL), mean ± SD	10.96±0.81	9-12
Hemoglobin (g/dL), mean ± SD	13.7±2.04	12-16
AST (IU/L), median (IQR)	148.5 (43.3-378.0)	0-32
ALT (IU/L), median (IQR)	101.0 (33.5-256.0)	0-33
LDH (IU/L), median (IQR)	443.0 (305.0-704.5)	135-214
CPK (IU/L), median (IQR)	222.0 (79.3-339.3)	0-170
ALP (IU/L), median (IQR)	65.5 (56.3-135.8)	35-105
GGT (IU/L), median (IQR)	51.0 (37.2-151.8)	6-42
Total bilirubin (mg/dL), median (IQR)	0.55 (0.40-0.70)	0.1-0.2
BUN (mg/dL), median (IQR)	15.0 (9.8-19.5)	6-20
Creatinine (mg/dL), median (IQR)	0.68 (0.61-0.88)	0.5-0.9
Procalcitonin (µg/L), median (IQR)	0.26 (0.12-0.65)	<0.05
CRP (mg/L), median (IQR)	7.1 (2.5-27.0)	0-5
PT (sec), median (IQR)	13.7 (12.4-15.8)	12-16.5
aPTT (sec), median (IQR)	33.0 (29.9-37.7)	25-36
INR, median (IQR)	1.0 (0.93-1.18)	0.8-1.2
D-Dimer (µg/L), median (IQR)	2052.0 (1051.8-3392.5)	0-500
Fibrinogen (mg/L), mean ± SD	3107.6±1276.73	2000-4000

In our study, the median IL-1β, IL-6, IL-8, TNF-α, HMGB1, and IL-10 levels were found to be 1173.6 (IQR: 783.0-1823.0) pg/mL, 69.9 (IQR: 56.8-133.1) pg/mL, 191.2 (IQR: 152.8-516.9) pg/mL, 129.5 (IQR: 104.9-270.8), 37.01 (IQR: 29.26-75.18), and 190.1 (IQR: 147.8-387.8) pg/mL, respectively (Table 4).

**Table 4:** Cytokine levels of CCHF patients. IL: interleukin; HMGB1: high-mobility group box-1; IQR: interquartile range; CCHF: Crimean-Congo hemorrhagic fever

	Total (n=30)
IL-1β (pg/ml), median (IQR)	1173.6 (783.0-1823.0)
IL-6 (pg/ml), median (IQR)	69.9 (56.8-133.1)
IL-8 (pg/ml), median (IQR)	191.2 (152.8-516.9)
TNF-α (pg/ml), median (IQR)	129.5 (104.9-270.8)
HMGB-1 protein (pg/ml), median (IQR)	37.01 (29.26-75.18)
IL-10 (ng/ml), median (IQR)	190.1 (147.8-387.8)

The patients' median SSI score was found to be 2.5 (1.8-5.5). In our study, three patients needed mechanical ventilation (MV). During the follow-up period, four patients died. The overall mortality rate was found to be 13.3% (Table 1).

**Figure 1:**
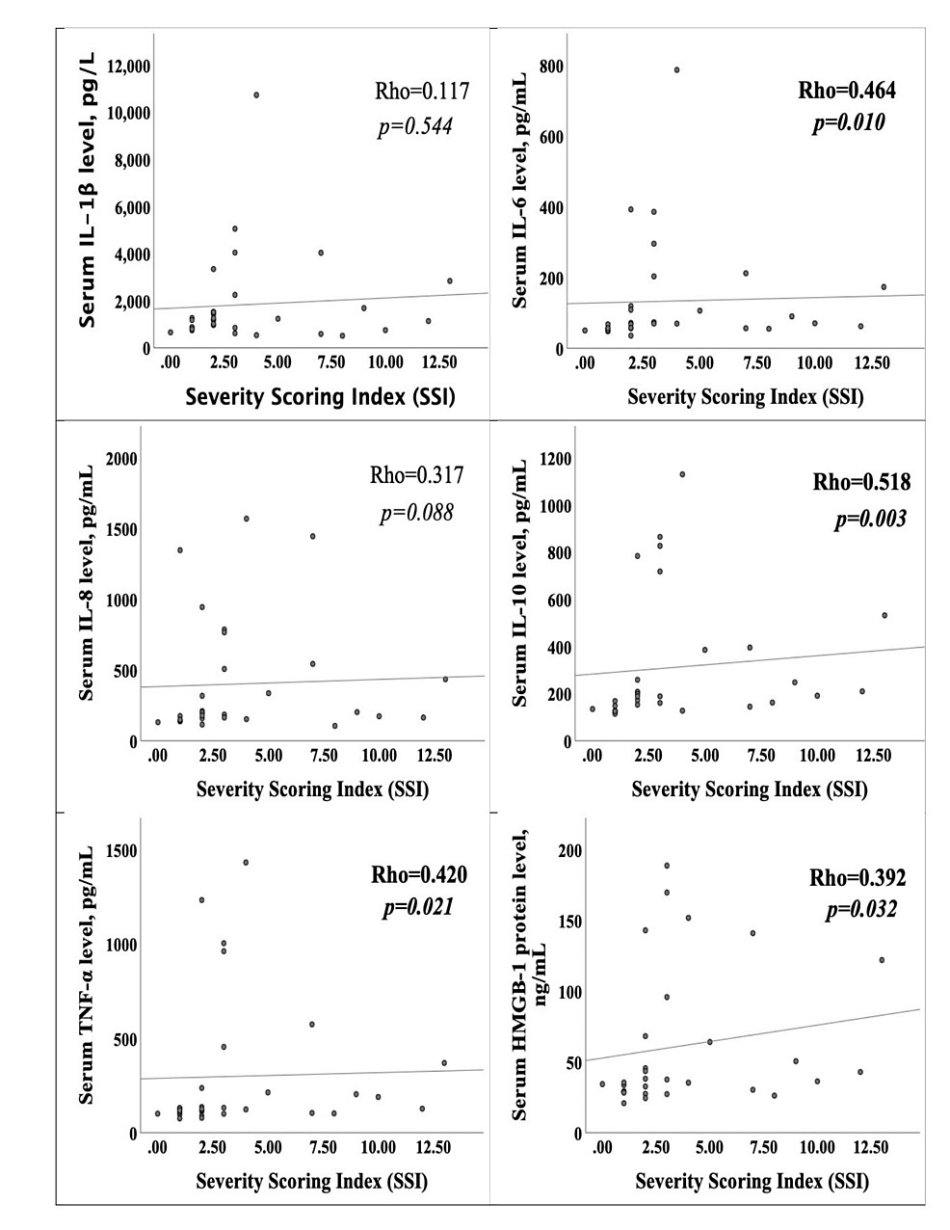
Relationship between inflammatory cytokine levels and Severity Scoring Index score

Correlation analysis between SSI score and serum inflammatory cytokines was evaluated. There was a moderate correlation between the patients' SSI score and TNF-α (r=0.420, p=0.021), IL-10 (r=0.518, p=0.003), and serum IL-6 (r=0.464, p=0.010) levels, and a weak correlation with serum HMGB1 (r=0.392, p=0.032). IL-1β and IL-8 levels and SSI score (p>0.05) gave no evidence of correlation (Figure 1).

**Figure 2:**
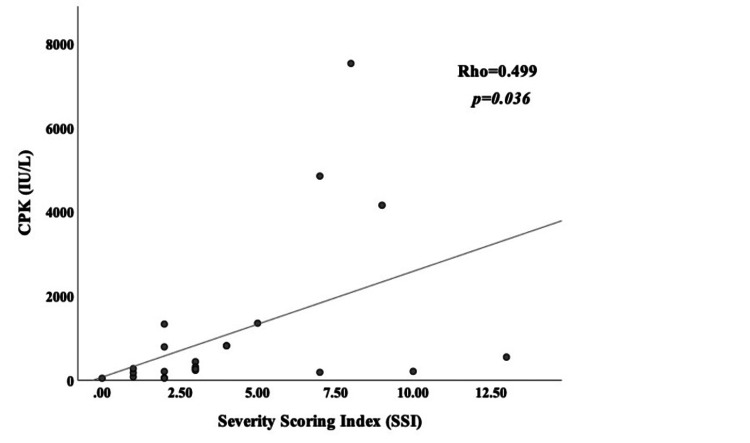
The relationship between CPK levels and SSI score CPK: creatine phosphokinase; SSI: Severity Scoring Index

According to the correlation analysis between serum CPK levels and SSI score, a moderate correlation was found between SSI score and CPK levels (r=0.499, p=0.036) (Figure 2).

## Discussion

Patients with CCHF fall along the clinical spectrum from being asymptomatic or mildly infected to being severely ill and in danger of imminent death. The time elapsed from the start of symptoms, viral load, comorbidities, and severity of the disease based on laboratory results directly affect patients’ rates of survival following admission. IL-10, IL-6, HMBG1, TNF-α, and CPK levels of CCHF patients evaluated upon admission to the clinic and SSI clinical score were found to be significantly related.

Within one to three days of a patient being bitten by a tick, a range of symptoms tend to rapidly present. These include neck stiffness, vomiting, fever, diarrhea, sore throat, myalgia, agitation, photophobia, and confusion. Pain and fullness are often seen in the abdomen (usually the right upper quadrant). Additional symptoms and signs can also present, e.g., petechia, lymphadenopathy, and tachycardia. Most patients in endemic areas have a subclinical infection at a rate of 88% [9]. Thrombocytopenia, coagulopathy, and leukopenia, as well as an increase in liver enzymes, are seen in the laboratory [10]. Approximately one-third of patients die five days or longer after the start of the disease due to bleeding or the failure of multiple organs [11].

The clinical findings detected upon examination at admission are as follows: hepatomegaly, occasionally peripheral lymph node enlargement, as well as splenomegaly (in about one-third of cases) [12,13]. Defined clinical features associated with patient mortality are gross hematuria, hematemesis, melena, and somnolence [8,14]. The mortality rate of 5-30% is usually defined by death occurring during the hemorrhagic phase. In our study, the most common clinical findings at the time of admission were fatigue (90%) and myalgia (63.3%). Bleeding was present in 10 (33.3%) study participants and seven (23.3%) had bleeding in more than one site. Our results are similar to the distribution of clinical findings in CCHF studies [14,16.17]

Depth of thrombocytopenia, prolongation of coagulation tests, and increased LDH and CPK levels indicate a poor prognosis [15,16]. In rare cases, macrophage activation syndrome with a severe increase in inflammatory cytokines exacerbates cytopenia [17-19].

Hemorrhagic fever severity is associated with hemophagocytosis, the result of TNF-α production, which promotes macrophage activation. In acting on the endothelium, TNF-α produces vasodilator substances, which have an antifibrinolytic effect. Recently, it has been shown to have a direct effect on endothelial cells via hantaviruses [20]. Tight junctions and conjoined junctions both maintain endothelial barrier integrity, and deregulation of either may result in increased vascular permeability [21]. Permeability increases as a result of vascular endothelial factors, which are an important component of endothelial cell tight junctions. However, as far as we know, there is insufficient evidence that CCHF infection directly acts on endothelial cells. IL-6, TNF-α, IL-12, IL-10, and (interferon) IFN-y levels, the serum pro- and anti-inflammatory cytokines, are seen to be higher in CCHF patients and raised in fatal CCHF cases [7,22]. This would indicate that capillary destruction, common in CCHF, is most likely the result of a combination of mechanisms that are host-induced and that arise due to the infection. The characteristic rash is caused by endothelial damage, which also contributes to hemostatic insufficiency. Similarities between septic shock and certain viral hemorrhagic fevers resulting from severe bacterial infections have been noted [23].

It is emphasized that the endothelial cells of the CCHF virus are damaged, particularly through cytokines released by monocyte-derived dendritic cells(moDC). Based on this hypothesis, Connolly-Andersen et al. reported in their study that while there was no increase in IL-1β and TNF-α levels in the infected endothelial cells of CCHF patients, there was an increase in IL-6 and IL-8 levels [5]. It was also reported that the addition of a TNF-α neutralizing antibody prevented moDC-mediated activation of endothelial cells. Therefore, it was emphasized that TNF-α is the key cytokine causing endothelial damage in CCHF [5].

To evaluate the prognosis of patients with CCHF during their admission, two different scoring systems, the SSI and the Severity Grading Score (SGS) were proposed. Both systems are estimated to have 96-100% sensitivity and 93-100% specificity in predicting the risk of poor prognosis [8,24]. These scoring systems were created for use in clinical trials in endemic countries, and they can help predict the clinical outcome of CCHF patients. SGS, one of these scoring systems, enables clinicians to determine whether the transfer of patients to a referral center is warranted or whether adequate care can be administered locally [5]. SSI, on the other hand, was developed to classify patients according to their mortality risks [8]. However, while clinical scoring systems do not account for it, where viral loads are in excess of 108 copies/mL, an independent association with poor prognosis has been observed [25].

In our study, the results demonstrated that SSI score was significantly associated with serum IL-6, TNF-α, IL-10 levels, HMGB1, and CPK values. No correlation was observed between serum IL-1β and serum IL-8 levels and SSI score.

A correlation between the severity of Ebola and dengue viruses (hemorrhagic fever viruses) for example, the degree of the pro-inflammatory response, and vascular permeability have also been reported. The key cytokines involved in the progression of the disease are as follows: TNF-α, IL-10, IL-6, and IL-1β [26]. IL-6 and other cytokines involved in liver proliferation produce Kupffer cells, or resident macrophages. Where liver injury is virus-induced, it leads to an increase in the expression of IL-6 in Kupffer cells or liver macrophages. Papa et al. performed a study in 25 patients with CCHF and 26 patients without any evidence of CCHF investigating the relationship between cytokine (TNF-α, sTNF-R, IL-6, and IL-10) concentrations and the severity of the disease. It was found that TNF-α concentration was associated with the severity of CCHF. However, they also found that IL-6 levels were elevated at a similar level irrespective of whether cases were mild or severe [22].

Saksida et al. reported that in patients where the clinical disease course was severe (mean: 83.6 pg/ml), IL-10 levels were notably elevated when compared to those patients in which the disease course was moderate (mean: 37.1 pg/ml) [7]. Similarly, IL-10 levels of patients with CCHF were found to be 190.1 (IQR: 147.8-387.8) pg/mL. The relationship between IL-10 levels detected in our results and clinical severity is similar to that seen in Saksida et al. In addition, Perez et al. conducted a prospective study on 28 patients with diagnosed dengue hemorrhagic fever to examine the levels of IL-12, IL-10, and RANTES (regulated on activation, normal T-cell expressed and secreted). IL-10 levels have been shown to be a strong inflammatory marker of thrombocytopenia and vascular damage [27].

In recent years, necrotic cells’ HMGB1 protein has been shown to contribute to a number of viral infections. The HMGB1 secreted is critical to the activation of many cells, including DCs, endothelial cells, and macrophages/monocytes, and serves to induce overexpression of a range of pro-inflammatory cytokines, including the ILs and TNF-α. The secretion of HMGB1 by autocrine or paracrine tends to further stimulate inflammatory responses through an increase in proinflammatory cytokine expression (e.g., TNF-a and IL-1β), which then stimulates the release of HMGB1. As the viral infection induces the release of HMGB1 and other proinflammatory cytokines, this in turn leads to an increase in the levels of HMGB1 and other proinflammatory cytokines released. This situation increases the significance of HMGB1 in viral infections. Rus et al. showed that HMGB1 could be a prognostic biomarker for the severity of CCHF infection and particularly a statistically significant difference between mild and severe patients [28]. However, findings have stated no relationship between HMGB1 levels and SSI. This difference was related to our study, which contained patients with mostly mild and moderate CCHF and a relatively small sample size.

Due to the sample feature of our study, there are inevitable limitations. No evaluation could be made between cytokine levels and clinical outcomes, as the study did not include a healthy control group, and cytokine levels were not obtained in intermittent follow-ups. In addition, the shorter time between the tick bite and post-symptom admissions of the patients in our study compared to the literature may have affected the clinical outcome. On the other hand, there are strengths of our study too. First, CCHF is an endemic disease in certain regions of Türkiye. Also, to our knowledge, there is no other study investigating cytokine (TNF-α, IL-8, IL-6, IL-10, IL-1β, and HMGB1) concentrations and SSI scores in CCHF patients. 

## Conclusions

Levels of IL-6, IL-10, TNF-α, and CPK measured during CCHF hospitalization are closely related to SSI scores and are important in terms of predicting poor clinical outcomes. While there was significantly a weak correlation between serum HMGB1 and SSI score, no statistically significant correlation was observed between serum IL-1β and serum IL-8 levels and SSI score. More studies with patients and groups of healthy volunteers are needed on this subject.
